# Determination of the Maximal Corrective Ability and Optimal Placement of the Ortho-SUV Frame for Femoral Deformity with respect to the Soft Tissue Envelope, a Biomechanical Modelling Study

**DOI:** 10.1155/2014/268567

**Published:** 2014-12-25

**Authors:** Petr V. Skomoroshko, Victor A. Vilensky, Ahmed I. Hammouda, Matt D. A. Fletcher, Leonid N. Solomin

**Affiliations:** ^1^Vreden Russian Research Institute of Traumatology and Orthopedics, 8 Baykova Street, Saint Petersburg 195427, Russia; ^2^Orthopedic Surgery Department, Al-Azhar University Hospitals, 10B Eltaka Street, El-Mabousin Buildings, 8th Area, Nasr City, Cairo 11371, Egypt; ^3^Department of Surgical Services, Dawson Creek & District Hospital, 11100-13th Street, Dawson Creek, BC, Canada V1G 3W8; ^4^Saint Petersburg State University, 7-9 Universitetskaja Street, Saint Petersburg 199034, Russia

## Abstract

Circular fixation according to the Ilizarov method is a well-recognised modality of treatment for trauma and deformity. One shortcoming of the traditional fixator is its limited ability to correct more than one plane of deformity simultaneously, leading to lengthy frame-time indices. Hexapod circular fixation utilising computer guidance is commonplace for complex multidimensional deformity but difficulties often arise with correction of femoral deformity due to bulkiness of the frame construct, particularly in proximal deformity and in patients of increased size. The Ortho-SUV frame is an innovative hexapod which permits unique customisation to individual patient anatomy to maximise tolerance and optimal range of deformity correction. We hypothesised that the optimal configuration and maximal degree of correction achievable by the Ortho-SUV frame can be biomechanically modelled and applied clinically. A study was constructed using Ortho-SUV and femoral limb models to measure deformity correction via differing frame constructs and determine optimal frame configuration to achieve correction in proximal, middle, and distal third deformities with respect to the soft tissue envelope. The ideal frame configuration is determined for correction of deformity in all locations of the femur with the maximal parameters of correction calculated whilst avoiding and mitigating soft tissue irritation from bulky frame construction.

## 1. Introduction

**Figure 1:**
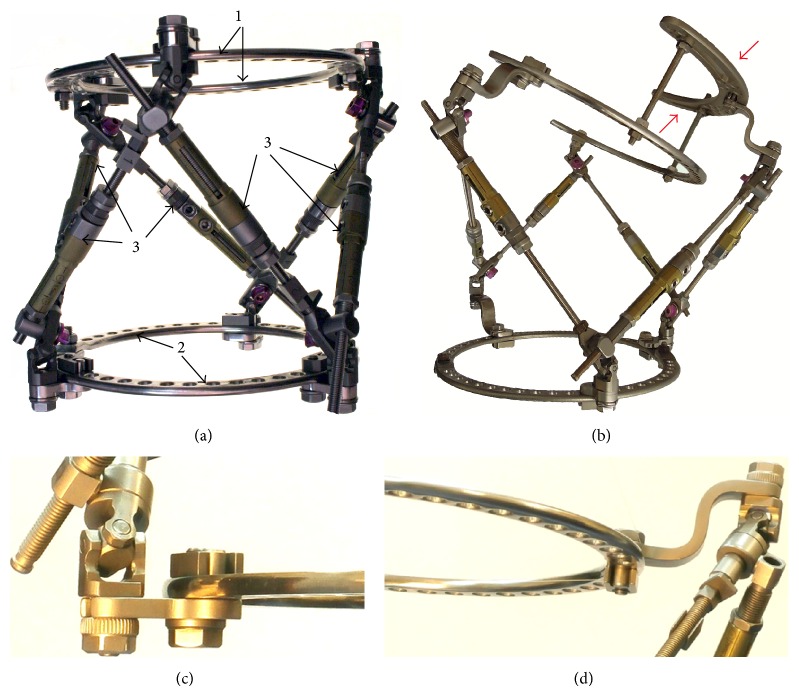
OSF. (a) Frame photo: 1: basic ring; 2: mobile ring; 3: universal reduction units; (b) configuration of the OSF with an additional stabilizing arch (indicated by arrows); (c) fixation of the strut to a ring by means of the straight plate; (d) fixed strut to a ring by means of the Z-shaped plate.

The Ortho-SUV frame (OSF) is a member of the hexapod circular external fixation devices, which utilise computer navigation for simultaneous three-dimensional correction [1]. The OSF (Figure 1) consists of two rings which are joined by a universal reduction unit comprising six telescopic struts connected to each of the rings directly or by means of plates. Where possible, additional stabilizing supports are added to increase the rigidity of fixation of bone segments [2]. The OSF has the ability to correct deformity in six axes in all planes with the aid of software and has been demonstrated to both improve the accuracy of correction and reduce correction time of deformity in the femur compared to the Ilizarov fixator [3].

No studies describe the biomechanics of the reduction capabilities of OSF in correcting femoral deformity. The purpose of this study was to assess differing OSF configurations to determine optimal design and potential for correction of femoral deformities and to precisely define the degree of correction achievable in the femur. We postulated that we could determine the maximal deformity correction potential of the OSF without impinging on the soft tissues. All frame configurations in this study were assembled according to the “Method of Unified Designation of External Fixation” [4].

## 2. Materials and Methods

OSF frame configuration and orientation with respect to the soft tissue at each level of a femur were carried out according to accepted rules of external fixation [5].

**Figure 2:**
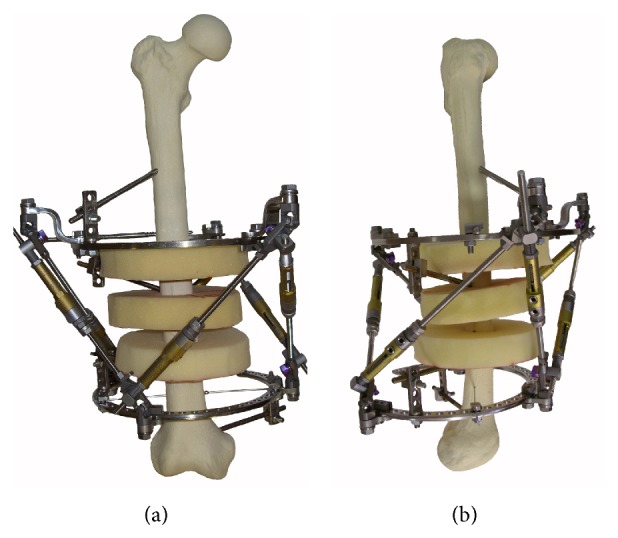
Models for the study of anatomical impingement of the OSF: (a) front view; (b) side view.

Models for the proximal, middle, and distal femoral thirds were assembled using plastic femurs. Soft tissue was modeled from polymer discs representing normal anatomy (Figure 2). Ring dimensions were selected so that the distance between the discs and the inner edge of the ring ranged from 3 to 4 cm. The form of disks and their arrangement relative to the bone were performed according to the “Atlas for the Insertion of Transosseous Elements Reference Positions” [2].

**Figure 3:**
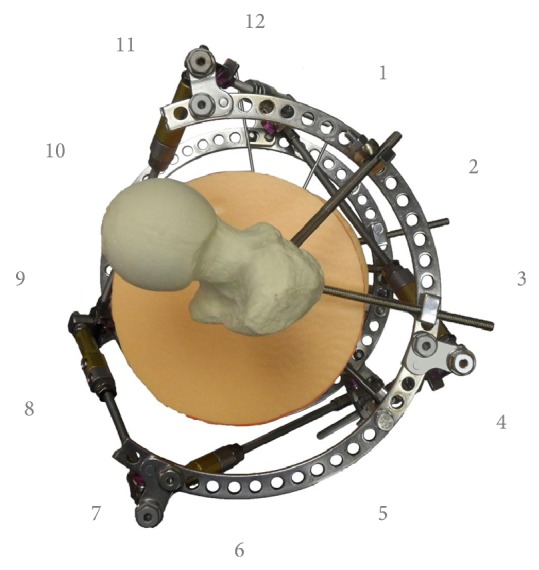
Method of designation of positions of strut-to-ring fixation using a clock-face analogy: strut number 1 is fixed in position 12, strut number 3 is fixed in position 7, and strut number 5 is fixed in position 4.

The experiments were divided into three series. Initially, we evaluated strut configurations for each of the three levels of the femur. Usually, incomplete rings and arches are used in frames for deformity correction in the femur, and so optimal equilateral triangular placement of struts has been difficult or impossible. Therefore, “unequilateral” strut configuration was necessary and thus the most effective configurations to obtain maximal corrective potential were investigated. With the OSF, strut position can be varied greatly and do not need symmetrical connection to the frame. To describe various strut configurations we described the circles of proximal and distal rings as a clock face and designated the strut's number and position on the ring in which the strut is fixed, with the 12 o'clock position being anterior in all cases (Figure 3).

**Figure 4:**
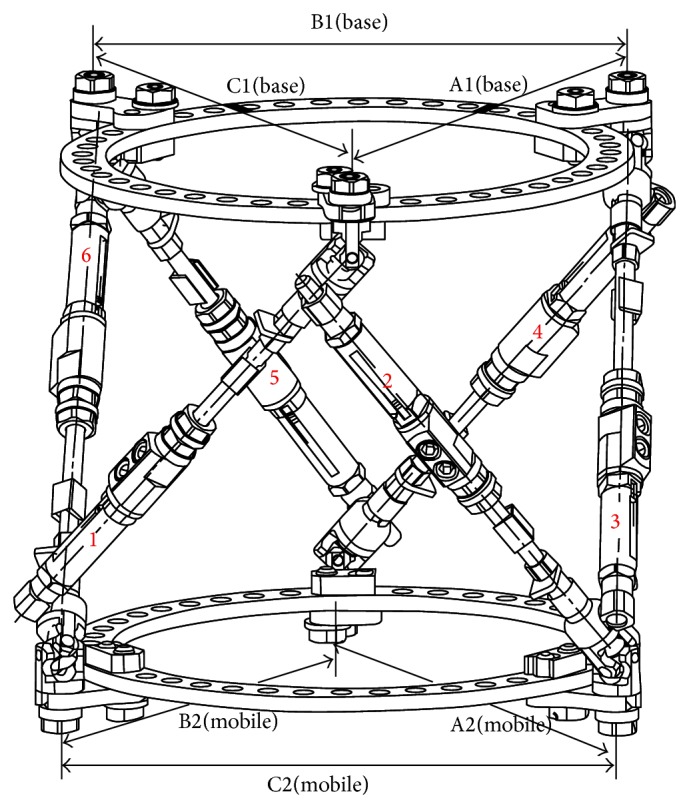
Orientation of struts relative to rings.

Each strut connects to an alternate ring in counterclockwise order, regardless of side; hence struts 1, 3, and 5 connect proximally and struts 2, 4, and 6 distally to rings, whilst the mobile segment of the strut connects to the base of the strut immediately preceding it; for example, strut 2 connects to the distal ring and proximally to the base of strut 1 (Figure 4). These produce two virtual triangles representing the base and mobile rings permitting accurate measurement of the computer driven correction parameters. Using the MUDEF clock face ring fixation position previously described in Figure 3 it can be seen that strut 1 affixes to position 12 on the proximal ring, strut 2 to position 2 on the distal ring, strut 3 to position 4 on the proximal ring, strut 4 to position 6 on the distal ring, strut 5 to position 8 on the proximal ring, and strut 6 to position 10 on the distal ring in this example.

For the proximal third of the femur 3 variants of OSF struts arrangement were analyzed as follows:

- Variant P1: strut number 1 in position 12, strut number 2 in position 9, strut number 3 in position 6, strut number 4 between positions 4 and 5, strut number 5 in position 3, and strut number 6 in position 1;
- Variant P2: strut number 1 in position 12, strut number 2 in position 9, strut number 3 in position 6, strut number 4 between positions 4 and 5, strut number 5 in position 3, and strut number 6 between positions 1 and 12;
- Variant P3: strut number 1 in position 12, strut number 2 in position 9, strut number 3 in position 6, strut number 4 in position 5, strut number 5 in position 2, and strut number 6 between positions 1 and 2.

For the middle third of the femur 4 variants of OSF struts arrangement were analyzed as follows:

- Variant M1: strut number 1 in position 12, strut number 2 in position 9, strut number 3 in position 7, strut number 4 in position 6, strut number 5 between positions 3 and 4, and strut number 6 between positions 1 and 2;
- Variant M2: strut number 1 in position 12, strut number 2 in position 9, strut number 3 in position 7, strut number 4 between positions 5 and 6, strut number 5 between positions 3 and 4, and strut number 6 between positions 1 and 2;
- Variant M3: strut number 1 in position 12, strut number 2 in position 9, strut number 3 in position 7, strut number 4 between positions 5 and 6, strut number 5 between positions 3 and 4, and strut number 6 in position 1;
- Variant M4: strut number 1 in position 12, strut number 2 in position 9, strut number 3 in position 7, strut number 4 in position 5, strut number 5 between positions 3 and 4, and strut number 6 in position 1.

For the distal third of the femur 5 variants of OSF struts arrangement were analyzed as follows:

- Variant D1: strut number 1 in position 10, strut number 2 in position 8, strut number 3 in position 6, strut number 4 in position 4, strut number 5 in position 2, and strut number 6 in position 12;
- Variant D2: strut number 1 in position 10, strut number 2 in position 8, strut number 3 in position 6, strut number 4 in position 4, strut number 5 between positions 1 and 2, and strut number 6 in position 11;
- Variant D3: strut number 1 in position 10, strut number 2 in position 8, strut number 3 between positions 6 and 7, strut number 4 in position 4, strut number 5 in position 2, and strut number 6 in position 12;
- Variant D4: strut number 1 in position 10, strut number 2 in position 8, strut number 3 in position 7, strut number 4 in position 4, strut number 5 between positions 1 and 2, and strut number 6 in position 12;
- Variant D5: strut number 1 in position 10, strut number 2 in position 8, strut number 3 in position 7, strut number 4 in position 4, strut number 5 between positions 1 and 2, and strut number 6 between positions 11 and 12.

The frame configuration showing the best results was tested in the second series of experiments to determine the optimal distance between the rings to provide best performance. Intercalary ring distances of 120 mm, 150 mm, and 200 mm were studied to assess magnitude of correction possible.

Having determined the optimal position of struts and the distance between the rings, the third series determined the effects of fixing the struts to the rings by means of Z-shaped instead of straight plates. For this, we serially replaced straight plates with Z-shaped plates in each strut and investigated the changes.

For each OSF model, struts were unlocked and all possible movements of the distal bony fragment relative to the proximal fragment were assessed as follows.

1. Translation in the frontal plane and in the sagittal plane: translation was directly measured between bone fragments.
2. Angulation in the frontal and sagittal plane: this angulation was measured on acquired digital images.
3. Rotational movement in the transverse plane: amount of rotation was determined using wires inserted in the proximal and distal segments and calculated via digital imaging.

**Figure 5:**
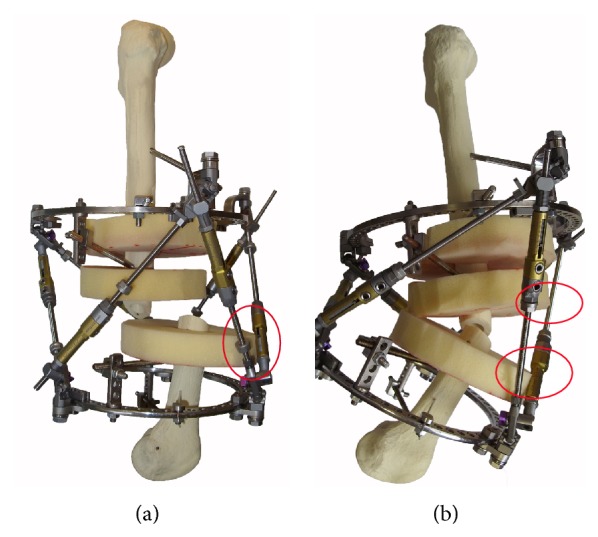
Limit of transverse (a) and angular (b) movement in a given model.

The limit of displacement in each case was reached when any strut touched the modeled soft tissue or achieved maximum possible length (Figure 5). Of note, distraction was not investigated since this parameter depends predominantly on the length of the threaded rods, which can be of any length in the OSF [6].

## 3. Results

The first series of experiments analyzed varying placement of the OSF struts for the three levels of the femur. Initial distance between the rings was 150 mm. This data (Table 1) demonstrates that, for the proximal third, option number 3 provided maximum angulation and internal rotation with a frontal plane arc of 71°, a sagittal plane arc of 77°, and a rotational arc range of motion of 50°. Also, it has nearly identical translation with option number 1 anteriorly, posteriorly, medially, and laterally and identical external rotation with option number 2 (*P* < 0.05).

For the middle third, option number 3 had a maximum translation laterally of 75 mm (*P* < 0.05). It was equal to other options (*P* > 0.05) in translation anteriorly (70 mm), posteriorly (80 mm), and medially (72 mm). Also, the values were similar in frontal plane angulation (93° arc) and in external rotation (35°). Option number 3 is second only in terms of internal rotation (35°) to option number 1 (50°), but it has superior performance in the majority of the remaining parameters.

For the distal third, option number 3 had maximum values of translation laterally (66 mm) and in recurvatum (40°) (*P* < 0.05). It had the same values in anterior (66 mm) and medial (85 mm) translation, in varus (35°) and procurvatum (35°) displacement, and also in both internal (27°) and external (30°) rotation (*P* > 0.05).

In the 2nd series of experiments, option 3 was selected as the best performing frame configuration, and the experiments were repeated with an intercalary distance between the rings of 120 mm and 200 mm and compared to the values for a distance of 150 mm. The results of the three femoral thirds showed that reducing the distance between the rings to 120 mm decreased all parameters significantly. Increasing the distance to 200 mm led to an increase in certain parameters (anterior and medial translation and varus angulation in the proximal third) and a decrease in others (posterior and lateral translation in all zones) (Table 2).

In the final series of experiments we studied the change in maximal parameter corrections of option number 3 when changing strut fixation to the rings with Z-shaped plates.  Table 3 summarises the results, which show that, for the proximal third of the femur, using a Z-shaped plate increases translation by 15–73%, angulation by 16–46%, and rotation by 26–35%. For the middle third, the amount of translation and angulation increases by 14–57% and 25–33%, respectively, with no significant increase in amount of rotation. For the distal third, there was 7–50% increase in the amount of translation, 14–43% in angulation, and 27–59% in the rotation.

**Figure 6:**
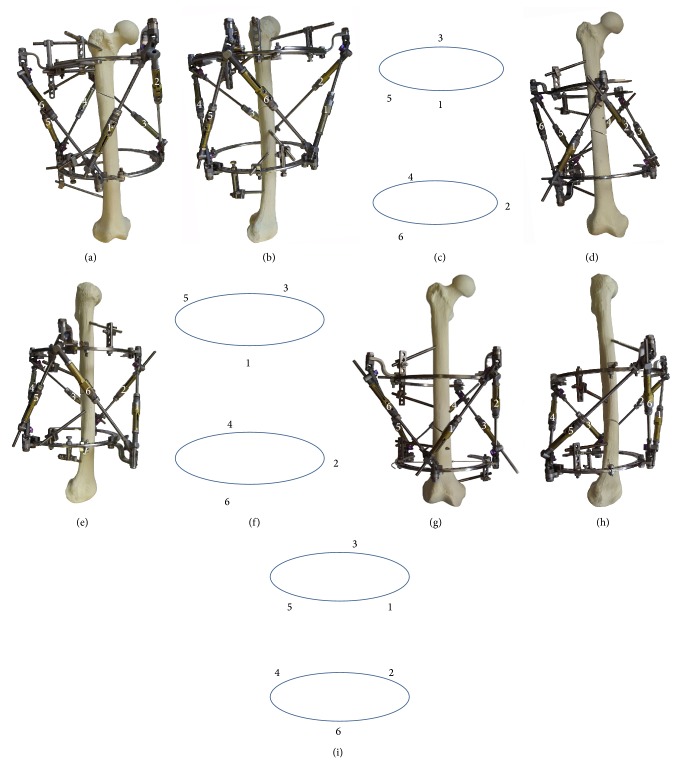
Optimum configuration of the OSF for correction of femoral deformity: (a), (b), and (c) proximal third right femur; (d), (e), and (f) middle third right femur; (g), (h), and (i) distal third right femur.

Thus, according to the results, it is clear that the best option providing maximal corrective ability within the femur is as follows (150 mm intercalary ring distance):

- in the proximal third of the femur (Figure 6): for the right femur, at the proximal ring, strut number 1 in position 12, strut number 3 in position 6, and strut number 5 in position 10; in the distal ring, strut number 2 in position 3, strut number 4 in position 7, and strut number 6 between positions 10 and 11; Z-shaped plates are used to fix struts number 1 and number 5;
- for the left femur: at the proximal ring, strut number 1 in position 12, strut number 3 in position 10, and strut number 5 in position 6; in the distal ring, strut number 2 between positions 10 and 11, strut number 4 in position 7, and strut number 6 in position 3; Z-shaped plates are used to fix struts number 1 and number 3;
- in the middle third of the femur (Figure 6), for the right femur: at the proximal ring, strut number 1 in position 12, strut number 3 in position 5, and strut number 5 between positions 8 and 9; in the distal ring, strut number 2 in position 3, strut number 4 between positions 6 and 7, and strut number 6 in position 11; Z-shaped plates are used to fix struts number 1 and number 5;
- for the left femur: at the proximal ring, strut number 1 in position 12, strut number 3 between positions 8 and 9, and strut number 5 in position 5; in the distal ring, strut number 2 in position 11, strut number 4 between positions 6 and 7, and strut number 6 in position 3; Z-shaped plates are used to fix struts number 2 and number 3;
- in the distal third of the femur, for the right femur: at the proximal ring, strut number 1 in position 2, strut number 3 between positions 5 and 6, and strut number 5 in position 10; in the distal ring, strut number 2 in position 4, strut number 4 in position 8, and strut number 6 in position 12; Z-shaped plates are used to fix struts number 1 and number 5;
- for the left femur: at the proximal ring, strut number 1 in position 2, strut number 3 in position 10, and strut number 5 between positions 5 and 6; in the distal ring, strut number 2 in position 12, strut number 4 in position 8, and strut number 6 in position 4; Z-shaped plates are used to fix struts number 1 and number 3.

## 4. Discussion

Femoral deformities and length discrepancy have multiple etiologies including trauma [7], congenital syndromes [8], metabolic conditions [9], and infections [10] and may lead to osteoarthritis, gait abnormality, and spinal disorders [11]. The Ilizarov method and its most recent iteration of hexapods have revolutionized the treatment of multiplanar deformity by enabling their simultaneous treatment [1–3].

OSF differs significantly in construction from other known hexapods. Its unique assembly permits the surgeon to address many kinds of femoral deformity [3]. In contrast to TSF [12], it does not require standard ring diameter or strut lengths and can be used easily with any available ring or arch. Furthermore, strut length is not limited and strut fixation to the rings may be customized with straight plates and Z-shaped plates (to provide more clearance in narrow frame segments) and direct fixation to the rings without using plates. This easily customizable assembly permits ready application to a wide variety of deformities and limb segments.

Critical appraisal of the biomechanics of the working space of the TSF compared to the Ilizarov frame concluded that paediatric application of the TSF was very limited due to constructional design [13]. The OSF does not suffer this shortcoming. Narrow segment correction such as foot deformity is likewise more easily accomplished by the OSF.

Our study has demonstrated the optimal frame configuration to maximize deformity correction in the femur and had quantified maximal correction possible in all axes of movement.

With an intercalary ring distance of 120 mm, movement in all directions decreased in the three femoral levels compared to a distance of 150 mm. This showed that, in the majority of experiments, the ability for fragment movement terminated due to the struts reaching minimum length and not due to contact of the struts with soft tissue. Furthermore, with this short distance, strut angle was often very low (≤30°) relative to the rings especially when using open rings for the femur [14]. This low angle limited the maximum achievable range of the strut. Thus, the reduced potential for correction by the OSF with an intercalary distance of 120 mm was due to constructional limitations of the frame.

Increasing the distance between the rings to 200 mm in the three femoral levels significantly increased the corrective potential of OSF; however this was limited in certain planes due to contact of struts with soft tissue around the femur. Furthermore, increasing the distance between rings leads to an increase in all dimensions of the frame which could reduce patient tolerance dependent on individual anatomical variation. Therefore, a distance of 150 mm between rings appears optimal for OSF assembly in all zones of the femur.

**Figure 7:**
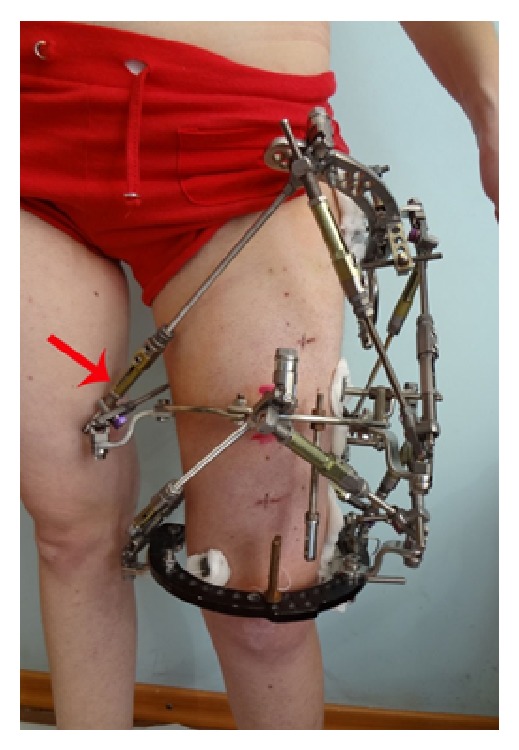
Medial impingement of Z-shaped plate.

By using Z-shaped plates to fix struts to the rings instead of the straight plates, correction potential of struts increased in the plane of the plate. However, the Z-shaped plate has the disadvantage of increasing the dimensions of the device which could lead to patient discomfort particularly at the interface between the medial and posterior thigh. Presence of Z-shaped plates on the medial side of the thigh causes impingement in adduction and in walking (Figure 7). Posterior positioning of Z-shaped plates interferes with supine positioning. Therefore, despite benefit gained from the Z-shaped plates, they should ideally be limited to application along the anterior and lateral aspects of the femur.

The results tables outline the maximal correction possible in all planes for varying configurations and can therefore be used as a guide for the surgeon to critically design and appraise frame configuration prior to application, ensuring that complete correction of preoperative deformity is achievable.

The Ilizarov method has greatly contributed to the fields of trauma, limb lengthening, reconstruction, and deformity correction. The mechanical properties of the Ilizarov frame in both traditional and hybrid forms have been demonstrated in the literature. Over the last decade, the hexapods have been introduced and embraced by the Trauma and Limb Lengthening communities. These frames have been used with great success for multiplanar deformity corrections, distraction osteogenesis, nonunion management, and fracture care, including the management of severe open injuries [1, 2, 15]. Treating complex deformity using conventional Ilizarov frames is time consuming [12]. Hence the prime benefit of hexapods is to permit correction of such deformities in a single step [3]. Furthermore, hexapods can be used as effectively as the Ilizarov for lengthening and trauma. The rigidity of the OSF appears greater in both standard and dynamised forms than the original Ilizarov frame [16].

This study demonstrates the biomechanical limits of correction achievable within the femur by the OSF, optimal frame design for maximal femoral deformity correction, and showcases its versatility for correction over and above existing and accepted hexapod fixators, as well as providing a useful tool for the surgeon to determine the feasibility and precise design of a frame in order to achieve complete correction of clinical femoral deformity when using the OSF.

## Figures and Tables

**(a):** 

Proximal third			
Movement	Option number 1	Option number 2	Option number 3
Translation in the sagittal plane	40 ± 1.1/65 ± 1.6	35 ± 0.9/45 ± 0.9	40 ± 2.0/65 ± 0.8
(anterior/posterior)^*^			

Translation in the frontal plane	43 ± 1.3/70 ± 1.1	30 ± 1.0/35 ± 1.1	48 ± 0.9/67 ± 1.1
(medial/lateral)^*^			

Angulation in the frontal plane	37 ± 1.6/24 ± 1.5	25 ± 1.5/25 ± 1.5	43 ± 0.6/28 ± 1.6
(varus/valgus)^†^			

Angulation in the sagittal plane	31 ± 2.4/36 ± 1.2	30 ± 0.8/20 ± 1.0	35 ± 0.9/42 ± 1.0
(procurvatum/recurvatum)^†^			

Internal rotation/external rotation^‡^	12 ± 1.2/19 ± 1.3	23 ± 1.6/24 ± 1.6	27 ± 0.6/23 ± 1.0

**(b):** 

Middle third				
Movement	Option number 1	Option number 2	Option number 3	Option number 4
Translation in the sagittal plane	58 ± 0.6/67 ± 0.6	70 ± 0.5/77 ± 0.4	70 ± 2.2/80 ± 0.8	70 ± 0.4/80 ± 0.5
(anterior/posterior)^*^				

Translation in the frontal plane	75 ± 0.7/52 ± 0.4	60 ± 0.3/60 ± 0.5	72 ± 1.1/75 ± 1.1	73 ± 0.3/50 ± 0.5
(medial/lateral)^*^				

Angulation in the frontal plane	43 ± 0.5/43 ± 0.3	45 ± 0.5/25 ± 0.4	45 ± 1.4/48 ± 0.8	45 ± 0.6/48 ± 0.7
(varus/valgus)^†^				

Angulation in the sagittal plane	15 ± 0.3/40 ± 0.2	38 ± 0.2/41 ± 0.3	40 ± 0.8/43 ± 0.9	20 ± 0.3/40 ± 0.2
(procurvatum/recurvatum)^†^				

Internal rotation/external rotation^‡^	50 ± 1.1/12 ± 0.6	35 ± 0.5/30 ± 0.4	35 ± 1.3/35 ± 0.9	30 ± 0.5/37 ± 1.6

**(c):** 

Distal third					
Movement	Option number 1	Option number 2	Option number 3	Option number 4	Option number 5
Translation in the sagittal plane	46 ± 0.4/60 ± 0.4	−5 ± 0.3/60 ± 0.4	66 ± 0.4/70 ± 0.6	70 ± 0.4/94 ± 0.5	67 ± 0.4/85 ± 0.6
(anterior/posterior)^*^					

Translation in the frontal plane	85 ± 0.2/60 ± 0.6	40 ± 0.3/41 ± 0.4	85 ± 0.5/66 ± 0.4	68 ± 0.3/45 ± 0.3	68 ± 0.4/45 ± 0.5
(medial/lateral)^*^					

Angulation in the frontal plane	35 ± 0.2/35 ± 0.2	20 ± 0.2/42 ± 0.2	35 ± 0.3/35 ± 0.2	37 ± 0.2/35 ± 0.3	35 ± 0.2/40 ± 0.4
(varus/valgus)^†^					

Angulation in the sagittal plane	34 ± 0.3/20 ± 0.2	35 ± 0.3/10 ± 0.4	35 ± 0.2/40 ± 0.2	35 ± 0.4/25 ± 0.2	34 ± 0.2/25 ± 0.3
(procurvatum/recurvatum)^†^					

Internal rotation/external rotation^‡^	27 ± 0.2/30 ± 0.2	10 ± 0.2/25 ± 0.2	27 ± 0.2/30 ± 0.2	10 ± 0.2/23 ± 0.2	14 ± 0.3/30 ± 0.2

^*^Values of translation measured in mm.

^†^Values of angulation measured in degrees.

^‡^Values of rotational measured in degrees.

**(a):** 

Proximal third			
Movement	Distance between rings	Distance between rings	Distance between rings
	of 120 mm	of 150 mm	of 200 mm
Translation in the sagittal plane	30 ± 1.9/35 ± 0.9	40 ± 1.1/65 ± 1.6	65 ± 0.8/33 ± 1.2
(anterior/posterior)^*^			

Translation in the frontal plane	15 ± 0.8/50 ± 1.1	48 ± 0.8/67 ± 1.1	63 ± 1.7/30 ± 0.6
(medial/lateral)^*^			

Angulation in the frontal plane	24 ± 0.9/13 ± 0.9	43 ± 1.6/28 ± 1.5	52 ± 1.6/30 ± 1.6
(varus/valgus)^†^			

Angulation in the sagittal plane	15 ± 0.7/40 ± 0.6	35 ± 2.4/42 ± 1.2	39 ± 0.6/43 ± 0.6
(procurvatum/recurvatum)^†^			

Internal rotation/external rotation^‡^	5 ± 0.6/5 ± 0.6	27 ± 1.2/23 ± 1.3	25 ± 0.8/26 ± 0.9

**(b):** 

Middle third			
Movement	Distance between rings	Distance between rings	Distance between rings
	of 120 mm	of 150 mm	of 200 mm
Translation in the sagittal plane	20 ± 0.3/45 ± 0.3	70 ± 0.7/80 ± 0.5	100 ± 0.4/70 ± 0.3
(anterior/posterior)^*^			

Translation in the frontal plane	50 ± 0.4/60 ± 0.4	72 ± 0.4/75 ± 0.2	100 ± 0.5/55 ± 0.4
(medial/lateral)^*^			

Angulation in the frontal plane	35 ± 0.5/27 ± 0.3	45 ± 0.6/48 ± 0.4	55 ± 0.6/60 ± 0.5
(varus/valgus)^†^			

Angulation in the sagittal plane	20 ± 0.2/30 ± 0.3	40 ± 0.4/43 ± 0.3	45 ± 0.3/53 ± 0.5
(procurvatum/recurvatum)^†^			

Internal rotation/external rotation^‡^	12 ± 0.3/25 ± 0.4	35 ± 0.5/35 ± 0.3	25 ± 0.4/37 ± 0.6

**(c):** 

Distal third			
Movement	Distance between rings	Distance between rings	Distance between rings
	of 120 mm	of 150 mm	of 200 mm
Translation in the sagittal plane	55 ± 0.3/54 ± 0.35	66 ± 0.4/70 ± 0.6	70 ± 0.5/55 ± 0.4
(anterior/posterior)^*^			

Translation in the frontal plane	23 ± 0.3/25 ± 0.4	85 ± 0.5/60 ± 0.4	43 ± 0.4/55 ± 0.3
(medial/lateral)^*^			

Angulation in the frontal plane	27 ± 0.3/27 ± 0.2	35 ± 0.3/35 ± 0.2	32 ± 0.5/38 ± 0.3
(varus/valgus)^†^			

Angulation in the sagittal plane	17 ± 0.2/25 ± 0.3	35 ± 0.2/40 ± 0.2	46 ± 0.4/45 ± 0.3
(procurvatum/recurvatum)^†^			

Internal rotation/external rotation^‡^	10 ± 0.2/16 ± 0.2	25 ± 0.2/30 ± 0.2	28 ± 0.7/40 ± 0.5

^*^Values of translation measured in mm.

^†^Values of angulation measured in degrees.

^‡^Values of rotation measured in degrees.

**(a):** 

Proximal third						
Movement	Strut number 1	Strut number 2	Strut number 3	Strut number 4	Strut number 5	Strut number 6
Translation in the sagittal plane	69 ± 0.6/	40 ± 1.1/	40 ± 1.5/	40 ± 0.3/	40 ± 0.3/	57 ± 0.3/
(anterior/posterior)^*^	64 ± 0.6	65 ± 1.6	75 ± 0.8	63 ± 0.4	63 ± 0.8	65 ± 0.5

Translation in the frontal plane	46 ± 0.4/	70 ± 2.3/	46 ± 2.4/	46 ± 0.3/	48 ± 0.3/	46 ± 0.3/
(medial/lateral)^*^	68 ± 0.5	65 ± 1.7	68 ± 2.1	68 ± 0.5	88 ± 0.3	68 ± 0.6

Angulation in the frontal plane	40 ± 0.4/	43 ± 1.6/	43 ± 1.3/	55 ± 0.5/	50 ± 0.4/	40 ± 0.4/
(varus/valgus)^†^	28 ± 0.3	24 ± 1.5	30 ± 0.8	25 ± 0.2	24 ± 0.3	30 ± 0.5

Angulation in the sagittal plane	33 ± 0.4/	35 ± 1.1/	32 ± 1.0/	35 ± 0.4/	35 ± 0.4/	29 ± 0.5/
(procurvatum/recurvatum)^†^	45 ± 0.6	40 ± 1.1	43 ± 1.4	45 ± 0.5	45 ± 0.4	45 ± 0.5

Internal rotation/	25 ± 0.4/	29 ± 1.5/	29 ± 1.8/	28 ± 0.3/	34 ± 0.5/	24 ± 0.5/
external rotation^‡^	31 ± 0.35	25 ± 1.5	25 ± 1.6	22 ± 0.3	22 ± 0.3	22 ± 0.3

**(b):** 

Middle third						
Movement	Strut number 1	Strut number 2	Strut number 3	Strut number 4	Strut number 5	Strut number 6
Translation in the sagittal plane	110 ± 0.7/	70 ± 1.2/	80 ± 1.5/	70 ± 0.5/	90 ± 0.5/	100 ± 0.5/
(anterior/posterior)^*^	80 ± 0.45	80 ± 1.1	80 ± 1.2	80 ± 0.6	80 ± 0.55	80 ± 0.5

Translation in the frontal plane	70 ± 0.35/	110 ± 1.5/	75 ± 0.9/	110 ± 0.6/	75 ± 0.5/	90 ± 0.5/
(medial/lateral)^*^	75 ± 0.5	73 ± 1.5	70 ± 0.8	77 ± 0.3	110 ± 0.5	72 ± 0.3

Angulation in the frontal plane	45 ± 0.4/	45 ± 0.9/	44 ± 0.9/	50 ± 0.3/	45 ± 0.3/	52 ± 0.4/
(varus/valgus)^†^	48 ± 0.3	53 ± 1.1	46 ± 0.9	45 ± 0.4	50 ± 0.3	55 ± 0.6

Angulation in the sagittal plane	43 ± 0.3/	40 ± 1.5/	40 ± 1.3/	40 ± 0.3/	45 ± 0.3/	50 ± 0.25/
(procurvatum/recurvatum)^†^	40 ± 0.2	45 ± 1.1	57 ± 1.1	40 ± 0.3	40 ± 0.4	45 ± 0.3

Internal rotation/	35 ± 0.5/	33 ± 0.8/	30 ± 1.5/	35 ± 0.45/	35 ± 0.5/	37 ± 0.6/
external rotation^‡^	40 ± 0.4	35 ± 0.8	35 ± 0.8	35 ± 0.3	35 ± 0.3	35 ± 0.3

**(c):** 

Distal third						
Movement	Strut number 1	Strut number 2	Strut number 3	Strut number 4	Strut number 5	Strut number 6
Translation in the sagittal plane	80 ± 0.6/	66 ± 1.1/	68 ± 2.1/	66 ± 0.6/	68 ± 0.4/	72 ± 0.3/
(anterior/posterior)^*^	80 ± 0.4	71 ± 1.3	72 ± 1.4	70 ± 0.4	75 ± 0.5	69 ± 0.5

Translation in the frontal plane	85 ± 0.6/	85 ± 0.9/	85 ± 0.9/	85 ± 0.2/	83 ± 0.3/	84 ± 0.3/
(medial/lateral)^*^	80 ± 0.7	88 ± 0.9	60 ± 0.8	80 ± 0.55	90 ± 0.4	70 ± 0.2

Angulation in the frontal plane	43 ± 0.3/	43 ± 1.3/	36 ± 1.1/	35 ± 0.3/	38 ± 0.3/	35 ± 0.4/
(varus/valgus)^†^	35 ± 0.3	35 ± 1.5	36 ± 0.9	40 ± 0.2	50 ± 0.3	35 ± 0.3

Angulation in the sagittal plane	37 ± 0.3/	35 ± 1.2/	35 ± 1.5/	38 ± 0.3/	33 ± 0.3/	37 ± 0.2/
(procurvatum/recurvatum)^†^	40 ± 0.2	38 ± 1.5	48 ± 1.3	40 ± 0.3	42 ± 0.3	35 ± 0.3

Internal rotation/	30 ± 0.2/	26 ± 0.9/	26 ± 0.7/	26 ± 0.5/	43 ± 0.4/	28 ± 0.5/
external rotation^‡^	32 ± 0.3	28 ± 1.2	30 ± 0.9	33 ± 0.2	38 ± 0.3	38 ± 0.3

^*^Values of translation measured in mm.

^†^Values of angulation measured in degrees.

^‡^Values of rotation measured in degrees.
